# Intention to Leave Emergency Medicine: Mid-career Women Are at Increased Risk

**DOI:** 10.5811/westjem.2020.5.47313

**Published:** 2020-08-21

**Authors:** Michelle D. Lall, Sarah M. Perman, Nidhi Garg, Nina Kohn, Kristy Whyte, Alexa Gips, Tracy Madsen, Jill M. Baren, Judith Linden

**Affiliations:** * Emory University School of Medicine, Department of Emergency Medicine, Atlanta, Georgia; † University of Colorado School of Medicine, Department of Emergency Medicine, Denver, Colorado; ‡ Hofstra/Northwell Health, Department of Emergency Medicine, Long Island, New York; § Northwell Health, Feinstein Institutes for Medical Research, Long Island, New York; ¶ Vituity/DeKalb Emergency Physicians, Emory Decatur Hospital, Emory Hillandale Hospital, Atlanta, Georgia; || University of Colorado School of Medicine, Department of Hospice & Palliative Medicine, Denver, Colorado; # Alpert Medical School, Brown University, Department of Emergency Medicine, Providence, Rhode Island; ** Perelman School of Medicine, University of Pennsylvania, Department of Emergency Medicine, Philadelphia, Pennsylvania; †† Boston University Medical Center, Department of Emergency Medicine, Boston, Massachusetts

## Abstract

**Introduction:**

Burnout is prevalent among emergency physicians and may cause physicians to consider leaving the practice of emergency medicine (EM). This study sought to determine whether there is a gender difference in reporting burnout and seriously considering leaving the specialty of EM, and secondarily to explore the factors reported as contributing to burnout.

**Methods:**

This was a secondary analysis of the 2014 American Board of Emergency Medicine Longitudinal Survey of Emergency Physicians. We used multiple logistic regression to determine which factors were associated with reporting serious consideration of leaving EM, when stratified by years in practice and adjusting for individual, departmental, and institutional factors.

**Results:**

The response rate was 82%, (n = 868); 22.6% (194) were female and 77.4% (664) were males; and 83.9% (733) White. The mean age of men responding was significantly higher than women (52.7±11.9 vs. 44.9±10.4, p<0.001). Overall, there were no significant gender differences in reporting having had serious thoughts of leaving EM in either unmatched or age-matched analyses. More women reported that burnout was a significant problem, while men more often were equivocal as to whether it was a problem. When stratified by years in practice, mid-career women had a seven-fold increase in the odds ratio (OR) of seriously considered leaving EM, compared to men of similar years in practice (OR 7.07, 95% confidence interval, 2.45–20.39). Autonomy at work, control over working conditions, fair compensation, personal reward, and a sense of ownership were factors associated with a lower rate of reporting considering leaving EM.

**Conclusion:**

Our findings suggest that the intention to leave EM is not more prevalent in women. However, mid-career women more often reported seriously considering leaving the specialty than mid-career men. Further research on the factors behind this finding in mid-career women in EM is needed.

## INTRODUCTION

Burnout, defined as “a state of emotional exhaustion, depersonalization, and a lack of sense of personal accomplishment,” is prevalent among physicians.^1^ In the medical literature, burnout is often measured using the Maslach Burnout Inventory (MBI),^1^ Oldenburg Inventory,^2^ or single-item measures of emotional exhaustion and depersonalization.^3^ However, prior work on emergency physicians (EP) demonstrated that self-reported burnout (as assessed by “have you thought you are experiencing burnout”) accurately predicted burnout as defined by MBI scores 72% of the time.^4^ Burnout in physicians is associated with many negative effects including decreased job satisfaction,^5^ an increase in intention to leave a job,^6^,^7^ decreased job productivity,^8^ increased medical errors and decreased patient safety,^9^,^10^ and substance use disorders.^11^ The prevalence of burnout in attending physicians across medical specialties is more than twice that observed in the general adult working population, with EPs reporting one of the highest burnout rates – between 48–70%.^12^,^13^,^14^

Prior studies reveal gender differences in reported burnout, with women reporting burnout at higher rates than men, yet little is known about gender differences in burnout among EPs specifically.^15^–^21^ In one study of internal medicine residency program directors, women had higher rates of emotional exhaustion and depersonalization.^18^ A study of American surgeons revealed that women suffered from higher rates of burnout than men and also had higher rates of emotional exhaustion, a factor that has been shown to be associated with a desire to leave clinical practice.^20^ Data on gender differences in burnout in emergency medicine (EM) is lacking.

Previous studies have shown that older age may be protective against burnout.^22^,^23^,^24^ Older age has also been shown to be a positive factor in EP job satisfaction.^25^ Since women in EM are often of a younger age and may also have more non-clinical and family responsibilities,^26^,^27^ these factors may contribute to higher burnout rates among mid-career women. Mid-career seems to be a particularly vulnerable time for female physicians.^17^,^28^,^29^,^30^ Additionally, the notable decline in women who rise to higher ranks of leadership and seniority in EM^28^,^31^ could be either a contributor to, or a result of, potentially higher burnout rates for women.

Physicians suffering from burnout are significantly more likely to leave healthcare^6^,^7^,^32^,^33^,^34^, and those who report an intent to leave is a strong predictor of actual departure.^33^ Prior to leaving healthcare, physicians often reduce their work hours and change their clinical work environment in an attempt to ameliorate burnout.^13^ Burnout and the attrition of physicians from healthcare is quite costly.^34^,^35^,^36^,^37^ Our primary objective was to determine whether there is a gender difference in reported serious consideration of leaving the field of EM. As a secondary objective, we sought to determine whether previously identified domains contributing to burnout are associated with burnout among EPs.

**Population Health Research Capsule**

What do we already know about this issue?

There is attrition of women in academic emergency medicine. Women are less likely to achieve senior leadership positions or promotions than their male counterparts.

What was the research question?

Is there a gender difference in reporting burnout and seriously considering leaving EM?

What was the major finding of the study?

Mid-career women had a seven-fold increased odds ratio of seriously considering leaving EM, compared to mid-career men.

How does this improve population health?

Diversity and longevity in the EM workforce are critical; the factors contributing to mid-career women seriously considering leaving EM need exploration.

## METHODS

### Study Design and Setting

This is a secondary data analysis of the 2014 American Board of Emergency Medicine (ABEM) Longitudinal Study of Emergency Physicians (LSEP). The ABEM LSEP is a 36-page questionnaire that was sent every five years to an ongoing cohort of EPs, from 1994–2014. A full text of the survey can be found on the ABEM website.^38^ This study was approved by the Emory University Institutional Review Board as an exempt protocol.

### Selection of Study Participants

**Figure 1:**
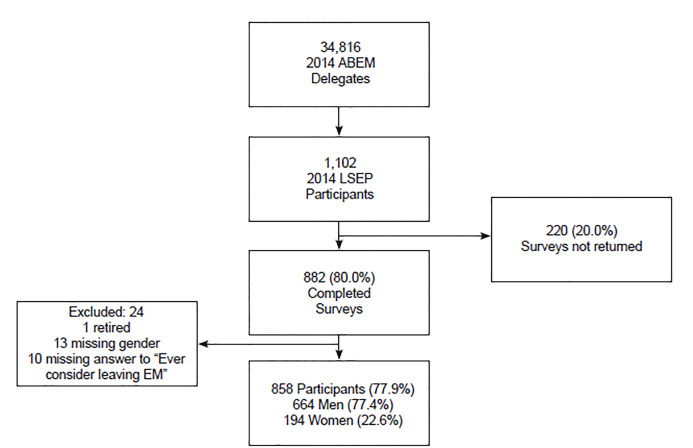
Flow diagram of participant exclusion in survey of emergency physicians. *ABEM*, American Board of Emergency Medicine; LSEP, Longitudinal Study of Emergency Physicians; *EM*, emergency medicine.

The first LSEP cohort identified in 1994 was selected via a stratified, random sampling of representative EPs within four different stages in the development of the specialty ensuring a representative sample of those who had completed EM residency and those who had not. Since that time, new cohorts were identified for inclusion every five years, until the final survey in 2014. Since 1999, all new cohorts are participants of Accreditation Council for Graduate Medical Education-approved EM residency programs. For the purpose of this study, all participants who responded to the 2014 questionnaire were considered for inclusion into this analysis. Subjects were excluded if they were not actively engaged in the practice of EM (ie, considered themselves fully retired), if they did not report their gender, or if they did not respond to the question “Have you ever seriously considered leaving the specialty of EM?”, as these variables were necessary for the primary outcome for this analysis (Figure 1).

### Study Outcomes and Variables

The primary outcome was to determine whether there was a gender difference in board-certified EPs having considered leaving the specialty of EM. To explore this question, we analyzed the question “Have you ever seriously considered leaving the specialty of Emergency Medicine” with a dichotomous answer (Yes/No). Serious consideration of leaving the specialty was used as a surrogate for burnout. Additionally, we hypothesized that a greater proportion of women would report burnout to be a problem in their everyday work experience. To answer this question, we used the query “How much of a problem is burnout in your day-to-day work for pay?” with 1 not being a problem, and 5 being a serious problem (five-point Likert scale).

The secondary outcome was to explore the prevalence of previously identified domains associated with burnout^19^,^21^,^39^,^40^,^41^ in this cohort of EPs. Specifically, what proportion of survey respondents who were seriously considering leaving the specialty of EM self-identified the following burnout domains: 1) workload: demands of the job exceed capability; 2) lack of control; 3) lack of perceived reward: more about recognition, less about salary; 4) lack of community: socially toxic environment, lack of fairness/equity, lack of respect; and 5) value conflict: a disconnect between values that give meaning to life and day-to-day work.^42^,^43^

### Statistical Analysis

De-identified data were provided to the research team. All analyses were carried out using STATA, v14.1 (StataCorp, College Station, TX). We used standard descriptive statistics to characterize the men and women who completed the survey. Categorical variables were compared using chi-squared tests, while we compared continuous variables using analysis of variance (ANOVA).

#### Analysis for Decision to Leave Emergency Medicine

To further explore the associations between gender and the intention to leave EM, we performed unadjusted logistic regression to determine whether there was an association between gender and the intention to leave EM. Subsequently, we also conducted multivariable logistic regression that accounted for years in practice. In review of the demographics within the cohort, male respondents were significantly older than the female respondents. To account for this, we created a 1:1 age-matched cohort of men and women in EM. Within this cohort, we explored the association between gender and the intention to leave EM via unadjusted analysis.

#### Analysis of Burnout in Day-to-Day Work

To explore burnout, we asked the question “how much of a problem is burnout in your day-to-day work for pay?” This question was reported as a five-point Likert scale, and we determined the proportion of respondents who considered leaving EM. The five-point Likert scale was then reconfigured into three categories with 1) burnout is a problem (score of 4 or 5); 2) burnout is not a problem (score of 1 or 2); and 3) ambivalent if burnout is or is not a problem (score of 3). Using these three categories, we used ANOVA to test for differences in gender and burnout. Next, we explored the association between gender and the decision to leave EM accounting for burnout and duration of time in practice using multivariate logistic regression.

The secondary objectives of this study were to explore the prevalence of variables identified as contributors to burnout in both male and female survey respondents who were considering leaving EM. These variables included the following: 1) autonomy at work; 2) compatible colleagues; 3) control over working conditions; 4) fair compensation; 5) personal reward; and 6) sense of ownership. The proportion of men and women who acknowledged the presence of these conditions in their current positions was reported using descriptive statistics. We used logistic regression to explore the association between each domain, gender and “seriously considering leaving EM” by including interaction terms between gender and each domain variable.

In all analyses, a p-value of 0.05 was considered statistically significant. We conducted analyses using STATA v14.1.

## RESULTS

The 2014 LSEP surveys were sent to 1102 EPs. A total of 882 physicians responded (80% response rate for all respondents, 82% for practicing physicians). We excluded 10 participants because they did not answer the primary study question, “have you seriously considered leaving the specialty of EM?” An additional 13 participants were excluded for not reporting their gender. Finally, we excluded one individual who did not answer any questions pertaining to practice, which we surmised meant he or she was retired. In total, the final cohort analyzed was 858 study participants (Figure 1). Of those included, 22.6% (n = 194) were women and 77.4% (n = 664) were men.

Table 1 describes the differences between the men and women who responded to this survey. Most notably, women who responded to the survey were younger than the men who were surveyed (44.9 ± 10.4 vs 52.7 ± 11.9, p<0.001), and a greater proportion of women had been practicing for 10 years or less in comparison to men (43.0% vs 22.9%). The younger age and shorter length of time spent in the field of EM was significant between men and women. The overall cohort was predominantly White, which did not allow for sufficient analysis of the effect of race/ethnicity in our findings.

In unadjusted analysis, there was no significant gender difference in the desire to leave EM (odds ratio [OR] 1.04, 95% confidence interval [CI], 0.74–1.43). Given the notable difference in age of men and women in our cohort, we used multivariable logistic regression to explore the association between gender and serious thoughts of leaving EM when accounting for years in practice (Table 2). When categorized into career state (early, mid, late), mid-career women (7–10 years of practice) had a seven-fold increase in the OR of “seriously considered leaving EM” compared to those in early stage careers (OR 7.07, 95% CI, 2.45–20.39) (Table 2). This trend persisted into late-stage career, where women had a higher likelihood of having serious thoughts about wanting to leave EM (Table 2). Due to the smaller proportion of women in the dataset, the CIs were large; nevertheless, we still observed a trend indicating women had a higher likelihood of considering leaving the specialty later into their careers in EM.

To account for the marked difference in age of men and women who responded to the survey, we also created an age-matched cohort of men and women. The matched cohort included 374 individuals (187 age-matched female-male pairs). In this age-matched cohort, the mean age of men and women was 45.0 ± 10.5 years. In this cohort, there was no association between gender and seriously considering leaving EM (OR 1.33, 95% CI, 0.87–2.04).

**Figure 2:**
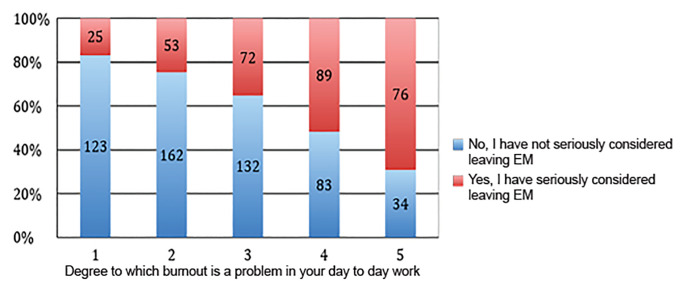
Association between burnout and considering leaving emergency medicine for all respondents (n = 852). Responses were pooled in a Likert scale of 0,1,2 (burnout is not a problem in everyday work for pay), 3 (ambivalent if it is or is not), 4,5 (burnout is a problem in everyday work for pay).

Among all survey respondents, we hoped to establish an association between considering leaving the field of EM and whether the participants reported burnout in their day-to-day work for pay. We found that an increasing desire to leave the field of EM was correlated with greater reporting that burnout heavily affected one’s day-to-day work for pay (Figure 2), and that the proportion of those reporting they had seriously considered leaving EM increased as self-reported burnout increased, with 76% of those in the highest burnout group reporting that they had considered leaving EM. Table 3 reports gender-based responses to the question of whether burnout is a problem, with a higher proportion of women stating it was a problem in comparison to men (37.3% vs 32.0%, p = 0.013). Unadjusted, burnout was significantly associated with seriously considering leaving EM (OR 5.2, 95% CI, 3.65–7.27). Adjusting for gender did not alter this association significantly (OR 5.2, 95% CI, 3.68–7.36).

Finally, we explored gender differences in the variables most associated with burnout. Table 4 outlines how frequently respondents reported having these conditions in their current work dichotomized by gender. There was no statistically significant gender difference except for women reporting less frequently “having control over working conditions” than men (50.3% vs 62.2%, p = 0.003). In addition, lack of autonomy at work, fair compensation, personal reward, and sense of ownership were associated with seriously considering leaving EM. When respondents reported having these conditions within their workplace, they were less likely to report having seriously considered leaving EM.

## DISCUSSION

In the population of physicians in this ABEM survey, there was no significant gender difference in the proportion of participants who had seriously considered leaving EM in the overall or age-matched cohort analyses. As of December 2014, ABEM had 32,238 diplomats: 56% male, 25% female, and 19% unknown gender (personal communication with ABEM in August 2018). In addition, we found that overall, 33.2% of all respondents in this cohort reported that “burnout was a significant problem in day-to-day work for pay,” which was lower than that reported in the literature.^4^,^13^,^44^ We found an association between burnout in day-to-day work for pay and consideration of leaving EM in both genders with mid-career women being significantly more likely to indicate that they had seriously considered leaving EM. Women were more likely to report that burnout was a significant problem, or that they were ambivalent as to whether it was or not, while men were more likely to answer that burnout was not a significant problem.

Finally, we found that women were more likely than men in this cohort to endorse “lack of control over working conditions.” The methodology of this study does not allow us to explain the reason for this observation. It is notable, however, that women are more likely than men to have a greater number of home and family responsibilities, and this finding may actually represent work-home conflict, and the lack of control over work-home responsibilities related to this conflict. Several studies have shown that physician and research scientist women have greater work-home conflict than male peers as they are often still responsible for a majority of household tasks, child caring and child rearing, and other unpaid work.^26^,^27^,^43^ Work-home conflict may be part of the reason why women are less likely to stay in academia and continue to lag behind their male counterparts in leadership positions and at the associate and professor ranks.^45^ In 2015, women made up 37.3% of EM residents and this number was relatively unchanged as compared to 2005.^46^ Women make up 43% of instructors, 37% of assistant professors, 24% of associate professors, and 17% of professors in EM.^47^ Additionally, there is a dearth of female chairs in EM. A 2015 report using American Academy of Medical Colleges (AAMC) faculty roster data revealed only 10 female chairs (11%) in EM as compared to 87 male chairs.^48^

Similar to prior literature, we found that women in mid-career practice may be especially vulnerable to considering leaving EM, compared to men at a similar point in career.^17^,^30^ It is important to consider potential contributors, and implications of our finding that women at mid-career were more likely to report having considered leaving EM. Since mid-career is the time when women often have more childcare and family responsibilities, such responsibilities may contribute to burnout. A study by Dyrbye et al found that mid-career physicians had the lowest satisfaction with their specialty choice and work-life balance, and had the highest rates of emotional exhaustion and burnout.^30^ Additionally, mid-career physicians were the most likely to plan to leave the practice of medicine for reasons other than retirement.^30^ Work-life concerns also prevent women from seeking promotion and leadership positions.^28^ The finding that mid-career women are more often considering leaving EM speaks to the importance of determining and incorporating best practices for flexibility to support women during this time in their career. Best practices for supporting women with family-friendly policies in academic EM have been identified, including flexible scheduling, emergency back-up childcare options, and policies that support healthy pregnancies and lactation areas.^49^ Ensuring that there is parity in advancement and compensation between women and men is also essential for retention of mid-career women.^49^ Many of these practices apply to both academic and community EM. If we can retain more mid-career women, not only will this lead to a more diverse community of practicing EPs but it will help to increase the number of women in senior EM ranks and positions, including at the associate and full professor ranks in academic EM, and as department leaders.

Historically, burnout was viewed as an individual-level problem and a sign of personal weakness, and as a result suggested interventions or modifying factors were focused on the individual level without consideration of organizational influences.^7^ As the body of literature on the prevalence of physician burnout continues to grow, there has been an increasing recognition of organizational and environmental causes for burnout. These causes include bureaucratic tasks (charting, paperwork), long work hours, electronic health records, lack of respect,^50^ insufficient compensation, lack of control/autonomy, feeling like a cog in a wheel, and profits over patients.^13^ Not surprisingly, we found that factors such as lack of autonomy at work, lack of control over working conditions, and lack of fair compensation all contributed to burnout, in both genders. The association between these previously established domains and our outcome of consideration of leaving EM supports the validity of our survey question on ever seriously considering leaving EM. Efforts to minimize these factors that contribute to burnout can be protective in maintaining the EM clinical workforce irrespective of gender and should be addressed in both community and academic settings.

## LIMITATIONS

There are several limitations to our study. First, the proportion of women in the sample was small (22%), which may have limited our ability to detect differences by gender. This is somewhat lower than the national percentage of women in EM as reported by ACEP (25%), ABEM (25%). (ABEM, Carmen Swinger, personal communication 11/22/2017), (ACEP personal communications 5/22/2018). The AAMC Group on Women in Medicine and Science, reported 37% of academic faculty in EM in 2015 were women.^31^ Second, the data only includes physicians who are currently practicing EM. We were limited in the conclusions we could draw about physicians who are no longer practicing or consider themselves fully retired, and what role burnout may have contributed to their reasons for leaving clinical practice.

In addition, the interpretation of the results of this survey is limited by the questions themselves as well as the format of the questions. This survey did not use a validated measure of burnout (MBI). Rather, we used the response to the question “How much of a problem is burnout if your everyday work for pay,”, and responses were dichotomized to a five-point Likert scale. This question has not been validated for identifying burnout but self-reported burnout in EPs has been shown to correlate with the MBI in a previous study.^4^ We also acknowledge that burnout is not a static condition, but rather often varies over time, as does consideration of leaving EM. Depending on the current situation at the time of the survey, the respondents’ recall of ever having considered leaving EM may vary. Additionally, we acknowledge that there are non-burnout related reasons that may result in someone responding in the affirmative to the question of seriously considering leaving EM.

As mentioned in the discussion, the age of the female cohort was younger than that of the male cohort. We accounted for this in our age-matched cohort, leading to a smaller sample size in the age-matched cohort, and found no difference in results. Finally, we were not able to perform any analysis related to minorities, given the very small number of respondents who identified as under-represented minorities (11% total).

## CONCLUSION

Mid-career women were more likely to have ever seriously considered leaving EM than mid-career men. Women were more likely to report that burnout was a significant problem in their day-to-day work for pay, while men were more likely to be ambivalent, or report that it was not a significant problem. Identifying and addressing the various factors that contribute to burnout and intention to leave the field of EM is critical, and emphasizing gender differences in these factors is necessary for retention and advancement. Mid-career women represent a particularly vulnerable group for increased rates of burnout and intention to leave the practice of EM, and intentional programming to support and promote this cohort is warranted.

## Figures and Tables

**Table 1:** Participant characteristics in the cohort of emergency physicians completing the American Board of Emergency Medicine survey stratified by gender.

Variable	Women	Men	P-value
Age (n=853)	44.9 ± 10.4	52.7 ± 11.9	<0.001
Race (n=851)			
White	82.4% (159)	87.2% (574)	0.086
Non-White	17.6% (34)	12.8% (84)	
# years in Emergency Medicine (n=697)			
0–1 years	12.3% (20)	3.4% (18)	<0.001
2–3 years	1.2% (2)	1.1% (6)	
4–6 years	22.1% (36)	12.2% (65)	
7–10 years	7.4% (12)	6.2% (33)	
11–20 years	31.9% (52)	22.1% (118)	
21–30 years	16.6% (27)	24.9% (133)	
>30 years	8.6% (14)	30.2% (161)	
Marital status (n=857)			
Married	74.7% (145)	88.5% (587)	<0.001
Divorced/Separated	7.8% (15)	3.9% (26)	
Single, cohabitating	5.7% (11)	2.9% (19)	
Single, living as single	11.3% (22)	3.9% (26)	
Widowed	0.5% (1)	0.8% (5)	
Children	1.5 (0, 2)	2.0 (1.5, 3)	<0.001
Current state of health (n=854) (Likert scale 1,2 = health concerns, 3,4 = no concerns)			
Exceptionally healthy	21.7% (42)	25.6% (169)	0.066
No health concerns	46.9% (91)	36.7% (242)	
Some minor health concerns	28.4% (55)	32.6% (215)	
Some serious health concern	3.1% (6)	5.2% (34)	
Clinical practice (n=846)	97% (84, 100)	90% (55, 100)	<0.001

**Table 2:** Multivariable analysis exploring the association between gender and “seriously considering leaving EM,” stratified by years in clinical practice.

Variable	OR	95% CI	P-value
Women (vs men)	1.23	0.82–1.84	0.309
Years in practice			
0–1 years	REF		
2–3 years	1.89	0.30–11.73	0.496
4–6 years	1.12	0.40–3.10	0.829
7–10 years	7.07	2.45–20.39	<0.01
11–20 years	2.61	1.02–6.64	0.045
21–30 years	4.96	1.94–12.70	0.001
>30 years	4.01	1.56–10.30	0.004

*EM*, emergency medicine; *OR*, odds ratio; *CI*, confidence interval; *REF*, reference.

**Table 3:** Self-reported assessment of burnout as a problem stratified by gender.

	Men (n = 656)	Women (n = 193)	P-value
Burnout IS NOT a problem (n=363)	45.4% (298)	33.7% (65)	0.013
Burnout IS a problem (n=282)	32.0% (210)	37.3% (72)	
I have no preference on if burnout is a problem or not (n=204)	22.6% (148)	29.0% (56)	

Responses were pooled in a Likert scale of 0,1,2 (burnout is not a problem in everyday work for pay), 3 (ambivalent if it is or is not), 4,5 (burnout is a problem in everyday work for pay).

**Table 4:** Availability of various work conditions stratified by gender and the association with “ever seriously considered” leaving emergency medicine.

Is each of the following work conditions available in your current position? (Y)	Women	Men	P-value (difference between men and women)	Univariate association with leaving EM OR (all gender)	95% CI	P-value
Autonomy at work (n = 851)	93.8% (180)	93.2% (612)	0.770	0.34	0.20–0.60	<0.001
Compatible colleagues (n = 849)	98.4% (188)	97.7% (643)	0.549	0.59	0.23–1.49	0.262
Control over working conditions (n = 847)	50.3% (97)	62.2% (407)	0.003	0.49	0.37–0.66	<0.001
Fair compensation (n = 845)	83.9% (162)	87.7% (572)	0.171	0.45	0.30–0.67	<0.001
Personal reward (n = 839)	85.4% (164)	90.3% (584)	0.058	0.33	0.21–0.52	<0.001
Sense of ownership (n = 845)	61.7% (119)	59.8% (390)	0.646	0.48	0.36–0.64	<0.001

*CI*, confidence interval; *OR*, odds ratio; *EM*, emergency medicine.
